# Plant adaptation to climate change

**DOI:** 10.1042/BCJ20220580

**Published:** 2023-11-23

**Authors:** Christine H. Foyer, Ilse Kranner

**Affiliations:** 1 School of Biosciences, College of Life and Environmental Sciences, University of Birmingham, Edgbaston B15 2TT, U.K.; 2 Department of Botany, University of Innsbruck, Sternwartestraße 15, 6020 Innsbruck, Austria

**Keywords:** cereal crops, drought, heat, oak forests, photosynthesis, seeds

## Abstract

Plants are vital to human health and well-being, as well as helping to protect the environment against the negative impacts of climate change. They are an essential part of the ‘One Health’ strategy that seeks to balance and optimize the health of people, animals and the environment. Crucially, plants are central to nature-based solutions to climate mitigation, not least because soil carbon storage is an attractive strategy for mitigating greenhouse gas emissions and the associated climate change. Agriculture depends on genetically pure, high-quality seeds that are free from pests and pathogens and contain a required degree of genetic purity. This themed collection addresses key questions in the field encompassing the biochemical mechanisms that underlie plant responses and adaptations to a changing climate. This collection encompasses an analysis of the biochemistry and molecular mechanisms underpinning crop and forest resilience, together with considerations of plant adaptations to climate change-associated stresses, including drought, floods and heatwaves, and the increased threats posed by pathogens and pests.

Plants provide over 80% of the food consumed by humans and they are the primary source of nutrition for livestock (Figure 1). Through photosynthesis, plants harvest sunlight to drive metabolism and generate biomass, and in doing so release molecular oxygen. The world's population continues to increase and is expected to approach 10 billion by 2050. A 50% increase in global food production by 2030 is required to meet the increasing demands for food. However, the current trajectory for the yields of many key crops (per unit area of land) is projected to be insufficient to nourish the increasing global population. At the same time, agriculture and related land-use change will generate roughly one-quarter of global greenhouse gas emissions.

**Figure 1.**
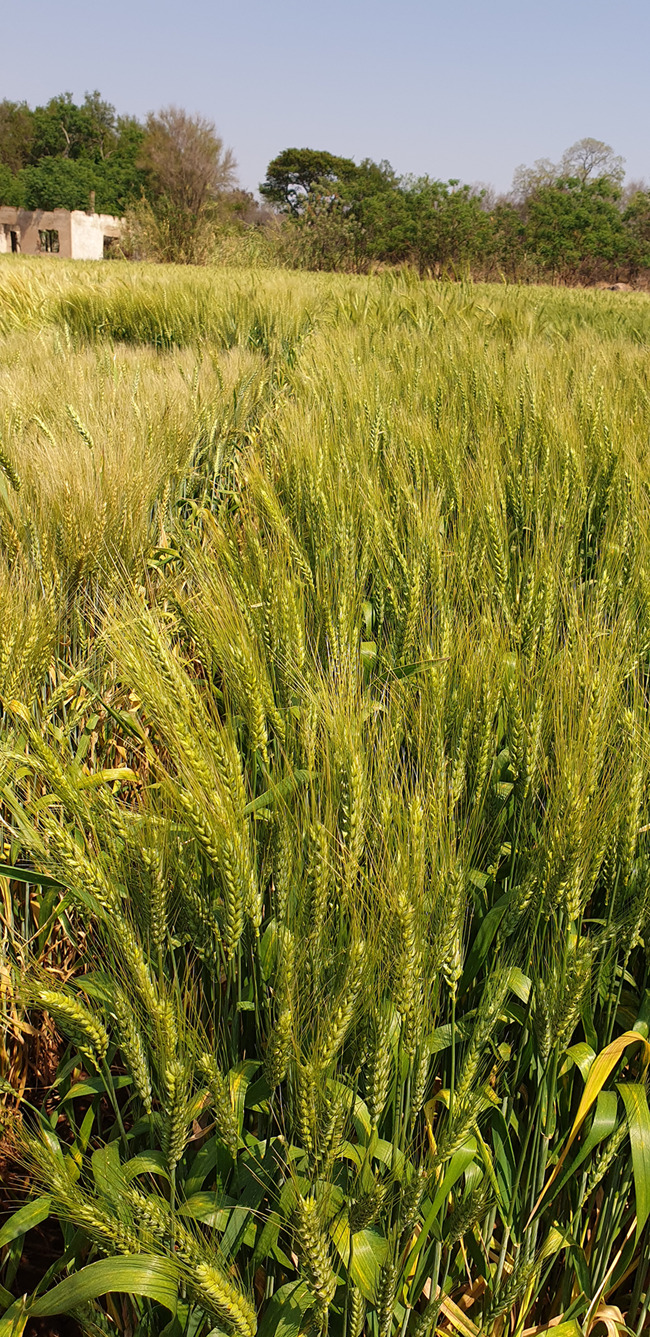
Plant production sustains life on Earth. Most extant life-forms, and humans especially, depend on photosynthetic carbon capture and oxygen production. However, driven by CO_2_ and other greenhouse gas emissions, climate change with its accompanying changes in temperature, more frequent droughts, heatwaves and floods is increasingly compromising plant productivity, often exacerbated by increasing plant vulnerability to pathogens and pests. In view of the need to feed an ever-increasing human population, the importance of nature-based solutions, including land restoration and reforestation, to climate mitigation needs to be recognized globally.

The impact of global warming and heatwaves on crop species has a direct impact on food security. Unfortunately, the pace of human-induced environmental change outstrips that of natural selection for many organisms, especially larger, slower reproducing, species that are often the cornerstones of an ecosystem. Moreover, climate change is predicted to alter the distribution and virulence of fungal pathogens, herbivores and other pests, as well as how plant hosts respond to these threats. Nevertheless, there are reasons for optimism. For example, there is unequivocal evidence that the Earth has become significantly greener over the past 30 years, with plants, particularly trees, producing greater leaf area as result of the global increases in carbon dioxide (CO_2_) and N deposition [1].

Root systems and their associated microbiota are the main source of C input into the soils, with subsequent sequestration by soil microbes. Governments across the world have initiated ambitious tree-planting policies and plant scientists have focussed on the development of new biotechnological strategies for sustainable crop production and forest resilience in a changing climate. Plants and their associated microbiota must adapt rapidly to the stresses that are predicted to accompany climate change, which is accompanied by increasing levels of CO_2_ and other greenhouse gases in the atmosphere. The rise in global temperatures as a result of increasing concentrations of greenhouse gases in the atmosphere is leading to global warming, together with increases in the probability and frequency of drought, floods and heatwave events, as well as threats posed by new pathogen and pest invasions. Together, the manuscripts that comprise this themed collection provide a comprehensive overview of the field encompassing the biochemical and other adaptations of plants, together with crop and forest resilience to climatic stress.

The authoritative review by Bach and Gojon [2] describes the biochemistry, molecular mechanisms and physiology that underpin the responses of root systems to increasing atmospheric CO_2_ levels. These authors discuss how root system size and architecture will be modified under high CO_2_ leading to improved C sequestration identifying important gaps in current knowledge. The effect of climate change on seed production and seed quality for global agriculture are assessed in the expert review by Bailly and Roldan [3]. Whether used directly for food and feed or through producing the next plant generation, seeds are the foundation of human life, from the food that we eat to the fibers in our clothing, as well as most of the products that we use in our daily lives. Moreover, successful germination and seedling establishment are important determinants of crop yields and plant survival in natural environments. Germination potential can be influenced by environmental conditions, which is at least in part, due to the effects the environment has on seed dormancy, i.e. the inability of a seed to germinate under optimal environmental conditions before certain cues have been perceived, e.g. chilling temperatures in the winter. Bailly and Roldan [3] provide unique personal insights and perspectives into the mechanisms that contribute to seed quality, and how seed biochemistry and physiology are affected by climate change, considering the prospects for new crop varieties and practices that will provide sustainable solutions for future agriculture.

Seed dormancy can be altered by the environmental conditions experienced by the mother plant, changing how the next plant generation spreads in time and space. This can have consequences to agriculture such as suboptimal crop germination, pre-harvest sprouting (e.g. in malting barley), or altered germination of weed plants. Moreoever, especially drought and elevated temperatures can result in seed abortion, causing significant economic losses as well as altering the quality of those seeds that stay on the mother plant, potentially inducing high levels of genome damage as well as altering seed storability. Hence, it is urgent to increase our knowledge of how climate change will alter seed quality not least because current seeds have not been selected for survival under hazardous and unpredictable weather conditions.

In their interesting paper on the mutagenic and growth inhibitory potential of DNA damage Waterworth and West [4] report that Arabidopsis seeds deficient in the chromosomal break repair factors DNA LIGASE 4 and DNA LIGASE 6 exhibited hypersensitivity to the effects of natural ageing, with reduced germination vigor (defined as the time taken for a seed lot to germinate) and seedling biomass relative to wild-type seeds. Data are presented showing that aged Arabidopsis seeds have increased levels of programmed cell death (PCD) in the root meristem, and this problem is increased in lines that are deficient in DNA double strand break repair. Moreover, data are presented showing that seed deterioration was accompanied by high levels of frameshift mutations and genome instability following germination.

Taylor et al. [5] dedicated their contribution to this themed collection to studying the role of the WHIRLY (WHY) family of DNA/RNA binding proteins. Compared with wild-type controls, germination was similar in unaged high-quality seeds of single and double mutants, but significant decreases in vigor and viability (defined as the ability to germinate, i.e. whether a seed is viable or dead) were observed in aged mutant seeds. Two mutants showed a significant delay in flowering, and produced more siliques per plant but with fewer seeds per silique than the wild type. Whereas imbibition (i.e. seed water uptake) of unaged high-quality seeds was accompanied by large increases in transcripts encoding proteins involved in oxygen sensing and hypoxia response, this ‘normal’ imbibition-induced transcriptome profile was disrupted by seed ageing. These authors conclude that WHY proteins are involved in the regulation of the responses to oxygen availability and hypoxia during imibibition, and that loss of WHY functions decreases the ability of Arabidopsis seeds to resist the adverse effects of seed ageing. Readers interested in seed biology and biochemistry are referred to the topical literature, including another themed collection of the Biochemical Journal entitled ‘Seeds’ and the references therein ([6–13]).

The topic of global warming on photosynthesis and crop production within the context of agriculture and agroecosystems is expertly reviewed in the article by Bernacchi et al. [14]. These authors expertly review the temperature-induced adaptations of plant biochemistry and metabolism that influence photosynthesis and biomass production, discussing the impacts and opportunities for enhancing the resilience of crops to global warming. The authors present key findings demonstrating that higher temperatures consistently reduce photosynthesis and crop yields even at high atmospheric CO_2_ levels. This timely and thought-provoking overview discusses the in-field heating techniques that are currently employed in studies designed to gain a better understanding of crop responses to warmer climates, as well as the adverse effects of season-long warming and heat waves. The article also contains a useful consideration of the effects of heat on atmospheric water vapor pressure deficits, photosynthesis and crop productivity, with a summary of current strategies being used to optimize the photosynthetic processes and enhance tolerance of increasing temperatures in order to minimize losses.

Photoprotection of the photosystem II reaction center (RCII) is addressed in the paper by Saccon et al. [15], whose work seeks to quantify the relative long-term effectiveness of photoprotection offered by the non-photochemical quenching component of chlorophyll a fluorescence quenching (NPQ) and the D1 repair cycle. These authors provide an estimation of the fraction of sustained decrease in RCII activity that is due to long-term protective processes and provide evidence highlighting the crucial role of constant and rapid D1 turnover for the maintenance of RCII efficiency. In addition, this paper provides a quantification of the contribution of a slowly reversible and protective NPQ component that does not impair RCII activity.

The study reported by Romero-Reyes et al. [16] provides a detailed account of the adverse effects of high field temperatures (over 30°C) on wheat photosynthesis and productivity in the Yaqui Valley, the main wheat producing region of Mexico. Working with the wheat flag leaves, photosynthetic capacity was assessed in 10 bread wheat genotypes grown under high field temperatures over two growing seasons. Data are presented showing that heat stress (HS) experienced during the grain-filling stage has a significant impact on grain yield leading to losses of almost 60%. The authors conclude that while HS reduced shoot biomass in all of the selected genotypes, three were able to maintain grain yields. This important finding suggested that these three genotypes could be introduced in this region for breeding heat-tolerant bread wheat cultivars.

The impact of climate change on the production of maize is compressively reviewed in the article by Yactayo-Chang et al. [17], with a key focus on the effectiveness of maize chemical defenses, including volatile organic compounds, terpenoid phytoalexins, benzoxazinoids, phenolics, and flavonoids. The authors discuss how these protective chemicals can be used to breed more climate-resilient maize varieties. This interesting review also considers the impact of multiple stresses on maize chemical defenses and discusses how exposure to individual or combined stresses can provoke the different metabolic responses.

Growth under high CO_2_ increases the demand for other resources such as nitrogen (N), phosphorus (P) and potassium (K) required to sustain growth and development. The mechanisms that plants use to perceive, take-up and assimilate these nutrients is described in the paper by Pahuja et al. [18], which focusses on the complex signaling pathways that are employed to avoid or overcome nutrient deficiencies. The main theme of this review is the intracellular calcium signaling system that facilitates NPK sensing and homeostasis. With a key focus on the sensors, transporters and transcription factors involved in signaling and responses, these authors provide a thought-provoking consideration of the calcium signaling components and pathways that underpin plant responses to NPK. The selective synthesis and degradation of proteins is the subject of the paper by Saini et al. [19], who report new results concerning the function of the Armadillo (ARM) repeat/U-box (PUB) protein family of E3 ligases. In particular, evidence is presented showing that AtPUB2 plays a role in oxidative stress tolerance by enhancing the activity of antioxidant enzymes.

The expert review by Smith and Luna [20] assesses the varied effects of elevated CO_2_ on plant immune responses to fungal pathogens, assessing results obtained in conflicting reports. These authors discuss how the effects of elevated CO_2_ on plant immune responses is specific to individual pathosystems. This detailed consideration concludes that there is no universal effect of elevated CO_2_ on plant immunity. The study reported in the paper by Sanchez-Lucas et al. [21] demonstrates that growth under elevated CO_2_ enhanced photosynthesis and aerial growth but reduced root length of oak (*Quercus robur*) seedlings. Crucially, high CO_2_ enhanced the susceptibility of the seedlings to the powdery mildew (PM) fungus (*Erysiphe alphitoides*). Moreover, data are presented showing that treatment with the elicitor β-aminobutyric acid (BABA) provided protection of the seedlings against PM under elevated CO_2_ and resulted in longer roots.

The collection features a wide range of comprehensive reviews and innovative research papers that demonstrate the central role of biochemistry and mechanistic adaptations in plant responses to climate change. The articles compiled not only provide novel results and perspectives but also encompases the reflections of experts on advances in current knowledge and the evolution of concepts in the field. This collection provides new insights into climate-driven shifts in plant productivity and plant–pathogen interactions. Taken together, these articles provides a greater mechanistic understanding of how plants respond to climate change, as well as how climate-driven shifts will modify agronomically- and environmentally important aspects of plant health and productivity.

## Open Access

Open access for this article was enabled by the participation of University of Birmingham in an all-inclusive *Read & Publish* agreement with Portland Press and the Biochemical Society under a transformative agreement with JISC.

## Abbreviations

HS: heat stress
PM: powdery mildew
RCII: photosystem II
WHY: WHIRLY
